# Efficient transformer integration in nnU-Net for liver tumor segmentation: an external validation study

**DOI:** 10.1186/s12880-026-02406-z

**Published:** 2026-05-22

**Authors:** He Cao, Li Tao, Fan Li, Xiaoping Pan

**Affiliations:** 1 Inner Mongolia Medical University, Hohhot, China; 2 International Mongolian Medicine Hospital of Inner Mongolia Autonomous Region, Hohhot, China; 3 International Mongolian Medicine Hospital of Inner Mongolia Autonomous Region, Inner Mongolia Medical University, Hohhot, China

**Keywords:** Liver tumor segmentation, NnU-Net, Convolutional transformer, Progressive unfreezing, External validation

## Abstract

**Background:**

Small and low-contrast liver tumors remain challenging targets for contrast-enhanced CT segmentation because of severe class imbalance and limited long-range contextual modeling in conventional CNN encoders.

**Methods:**

We developed OF-TransUNet, a minimalist hybrid that differs from parameter-heavy TransUNet-style variants by inserting a single lightweight mid-level Conv-Transformer block at encoder stage 3 (×8 downsampling) within an otherwise unchanged 2D nnU-Net, together with an output-focused progressive unfreezing schedule intended to improve adaptation stability. This design was motivated by unstable and poorly reproducible optimization observed under immediate full fine-tuning in internal ablation experiments. A public dataset (*n* = 104) was used descriptively for architecture and schedule selection. The pre-specified primary endpoint was the external per-patient tumor Dice difference versus a standardized 2D nnU-Net baseline on an independent cohort (*n* = 42). A pre-defined secondary lesion-level analysis focused on medium-sized tumors (10–50 mm).

**Results:**

On the external cohort, OF-TransUNet showed a numerically higher per-patient tumor Dice than nnU-Net (0.2788 ± 0.2575 vs 0.2400 ± 0.2426), with a mean paired difference of +0.0388 (95% CI 0.0029 to 0.0748). Because paired differences were non-normal, the pre-specified Wilcoxon signed-rank test was retained as the primary inferential analysis and was borderline (*p* = 0.0553); a supplementary paired t-test yielded nominal significance (*p* = 0.0347). In the pre-defined medium-lesion analysis (10–50 mm; *n* = 84), detection increased from 0.190 to 0.286 at the pre-specified 10% overlap threshold (McNemar exact *p* = 0.021; GEE OR 1.97, 95% CI 1.15–3.35). Post hoc sensitivity analyses using 5% and 15% overlap thresholds preserved the directional advantage. Relative to baseline, OF-TransUNet increased parameters by 8.4% and FLOPs by 18.3%, with no measurable latency penalty and only minimal memory increase.

**Conclusions:**

In this single-center external validation cohort, a single mid-level Conv-Transformer insertion plus output-focused progressive unfreezing was associated with a numerically higher per-patient tumor Dice and a statistically supported improvement in the pre-defined medium-lesion detection analysis, while preserving a lightweight computational profile. Because the pre-specified non-parametric primary patient-level analysis was borderline and did not reach conventional significance, the Dice finding should be interpreted cautiously. Tumor HD95 was numerically higher in OF-TransUNet, indicating a possible recall-boundary trade-off that requires further boundary-focused evaluation. Overall, this minimally invasive modification supports further multi-center validation rather than definitive claims of superiority. A supplementary controlled benchmark on the public LiTS dataset, limited to a single pre-fixed validation fold because only 131 public cases have released annotations for local evaluation, provided directionally consistent contextual evidence under an identical pipeline.

**Supplementary Information:**

The online version contains supplementary material available at 10.1186/s12880-026-02406-z.

## Introduction

Accurate liver and tumor CT segmentation supports quantitative volumetric assessment, treatment planning, and longitudinal response tracking [1], [2, 3]. Publicly available annotated hepatocellular carcinoma (HCC) datasets facilitate methodological benchmarking and reproducibility efforts [4]. The substantial and growing global burden of liver cancer reinforces the clinical imperative for robust and transparent automated segmentation tools [5, 6]. Deep learning-based automated liver tumor segmentation and classification approaches have demonstrated promising potential in clinical workflows [7].

Methodologically, progress has advanced from convolutional encoder–decoder baselines to hybrid designs. Convolutional architectures, including diverse U-Net derivatives and automated configuration frameworks, have delivered strong multi-scale feature extraction and generalization across tasks [8–14]. MLP-based architectures such as UNeXt have further explored rapid segmentation with reduced computational overhead [15]. Transfer learning paradigms inspired by unified text-to-text transformers have influenced multi-task medical imaging frameworks [16]. Nonetheless, constrained local receptive fields can limit global context modeling, contributing to missed or fragmented delineations for subtle lesions, while emerging Transformer and mixed CNN–Transformer variants seek to incorporate long-range dependencies with self-attention mechanisms [17, 18]. UNetFormer has demonstrated the effectiveness of unified vision transformer frameworks with pre-training strategies for 3D medical image segmentation [19]. Parameter-efficient fine-tuning strategies offer promising avenues to balance expressiveness and computational cost [20], and foundation models such as Segment Anything Model (SAM) have shown potential for zero-shot generalization in medical image analysis [21], though domain adaptation remains challenging. Emerging paradigms leveraging frozen pre-trained models for zero-shot learning in medical data analysis [22] suggest potential for parameter-efficient adaptation strategies, which motivate our progressive unfreezing approach. 3D TransUNet has extended transformer-based architectures to volumetric medical imaging tasks [23].

Persistent challenges include small or low-contrast tumors, severe class imbalance, heterogeneous enhancement patterns, anisotropic/thick clinical slices, peri-vascular adjacency, and cross-center domain shifts—all of which can degrade boundary fidelity and optimization stability [17, 18, 24–27]. Transfer learning strategies, including nnU-Net-based frameworks with ROI tokenization and cross-task attention, have been explored to address domain-specific challenges in cancer staging [28]. Large-scale foundation models such as SAM [29] and its medical adaptations [30] offer promising avenues for universal segmentation, yet require careful domain-specific fine-tuning. Reproducibility is further complicated when multiple architectural and training factors (stacked attention blocks, multi-branch encoders, large fusion heads, extensive pretraining, or aggressive fine-tuning) change simultaneously, obscuring causal attribution of reported gains [24–27].

Recent attention-enhanced and semantic guidance hybrids introduce dense channel–spatial and multi-gated mechanisms to narrow the semantic gap and strengthen cross-scale fusion [31, 32]. Fusion-based CNN–Vision Transformer (ViT) models emphasizing interpretability and multi-backbone integration extend this hybridization trend but may increase redundancy and computational complexity [33]. Furthermore, standard TransUNet variants typically stack multiple Transformer layers at the bottleneck [2, 11], which significantly increases parameter count and can complicate optimization on limited medical datasets. Conversely, emerging lightweight hybrid or MLP-based architectures often achieve efficiency by redesigning the network from scratch [8, 15], which abandons the highly optimized, self-configuring pipelines of established frameworks like nnU-Net [3] and obscures the exact source of performance gains. In contrast, we propose OF-TransUNet (Output-Focused TransUNet), a minimalist and causally isolatable strategy that inserts a single lightweight Conv-Transformer block at the mid-level encoder stage (×8) into an otherwise unmodified 2D nnU-Net and adopts an output-focused progressive unfreezing design, in which output-proximal components are prioritized during staged release to improve early optimization stability while preserving inference simplicity. A focused efficiency analysis (baseline vs single-block insertion) later confirms that the modification maintains a lightweight computational footprint without introducing a latency penalty. A public HCC dataset (*n* = 104) is used descriptively for architectural and schedule selection [4]; an independent cohort (*n* = 42) is reserved a priori for a single primary endpoint (tumor Dice improvement) under a standardized baseline protocol. Systematic ablations examine insertion depth and unfreezing regimes. Medium-sized (10–50 mm) lesions were designated for a pre-defined secondary analysis because large-scale cross-anatomy mining demonstrated a high prevalence of lesions within this diameter range [34], while benchmarked liver tumor segmentation performance exhibits a size-dependent improvement curve positioning medium lesions as an intermediate challenge between frequently missed very small nodules and saturating performance on large masses [35]. Our contributions are threefold: (1) a controlled single-point Conv-Transformer insertion within an otherwise unchanged 2D nnU-Net, enabling clearer attribution of architectural effect; (2) an output-focused progressive unfreezing strategy designed to improve adaptation stability during Transformer integration; and (3) external validation and comparative benchmarking under a standardized nnU-Net v2 pipeline, complemented by descriptive internal ablations of insertion depth and training strategy.

Because the LiTS benchmark remains one of the most widely used public references for liver tumor segmentation in contrast-enhanced CT, we additionally performed a supplementary controlled experiment on the public LiTS annotated cases to provide contextual validation of the proposed architecture. However, direct cross-study comparison on LiTS is methodologically difficult because published results differ substantially in split strategy, dimensionality (2D vs 3D), preprocessing, optimization schedule, and inference configuration. Therefore, LiTS-based evidence is presented in two layers: (1) a controlled within-pipeline supplementary benchmark using a pre-fixed public validation fold, and (2) a descriptive contextual summary of representative published LiTS studies. This distinction is intended to ensure that architectural effects are not conflated with pipeline heterogeneity, while still providing contextually relevant external benchmark evidence [35–37].

## Methods

### Study design

This retrospective methodological study developed and compared convolutional and hybrid Transformer–convolutional architectures for automatic voxel-wise liver and liver tumor segmentation on contrast-enhanced CT. Internal development experiments (public dataset, *n* = 104) were used strictly for descriptive architecture and training strategy selection. The pre-specified primary endpoint was the **per-patient** tumor Dice improvement of the proposed OF-TransUNet over the baseline 2D nnU-Net on an independent external validation cohort (*n* = 42). A comprehensive benchmark comparison was then conducted between OF-TransUNet, the nnU-Net baseline, and five other U-Net variants to contextualize its performance. To prevent data leakage, all architectural and training decisions (insertion stage, unfreezing schedule) were finalized based solely on internal development data (*n* = 104) before accessing the external validation cohort. The external cohort (*n* = 42) was reserved exclusively for one-time evaluation of the pre-specified primary endpoint (tumor Dice improvement) and secondary endpoint (medium-lesion detection rate). No hyperparameter tuning or model selection was performed on the external cohort.

### Datasets

#### Public development dataset (HCC-TACE)

We used the publicly released Multimodality Annotated Hepatocellular Carcinoma (HCC-TACE) dataset (104 cases) containing multi-class annotations (liver, tumor, intrahepatic vessels, abdominal aorta) on pre-treatment contrast-enhanced CT. Only liver and tumor labels were retained; vessel and aorta labels were ignored.

Phase usage followed a predefined hierarchy: the portal venous phase was selected; if the portal phase was unavailable, the arterial phase was used. Vendor diversity (GE, Siemens, Philips) introduced acquisition heterogeneity.

#### External validation cohort

An independent cohort of 42 liver cancer cases was retrospectively collected. Inclusion criteria: (1) contrast-enhanced CT findings consistent with liver cancer, (2) age ≥ 18 years, (3) complete triphasic imaging without severe motion or metal artifacts. Exclusion criteria: (1) incomplete clinical or imaging data, (2) corrupted DICOM series. All malignant liver lesions were treated uniformly as “tumor” without subtype stratification.

Imaging protocol: Triphasic contrast-enhanced CT on a 64-slice GE scanner; 80 mL iodinated contrast at 3.0 mL/s; arterial, portal venous, and delayed phases at 30 s, 60 s, and 180 s post-injection. Parameters: 120 kVp; 260–280 mAs; rotation 0.6 s; pitch ≈1.0; soft-tissue reconstruction kernel; field of view 320–400 mm; matrix 512 × 512; reconstructed slice thickness 5.0 mm. All external-cohort cases had portal venous phase data available, and this phase was used for model inference throughout. Arterial-phase images were reviewed only as an auxiliary visual reference for tumor-boundary assessment and were not used as fallback inference input. All images were de-identified.

#### Dataset split and case accountability

The public HCC-TACE collection contained 105 CT studies. One case (HCC_089) was excluded because its released segmentation files could not be reliably mapped to a valid liver-phase CT series: the mask loaded as empty after matching available SeriesInstanceUID metadata, and the referenced series directory (e.g., “6.000000-Recon 3 LIVER 2PHASE CAP-73419”) did not contain axial slices covering the liver region. This case was therefore treated as having a missing/invalid annotation and removed a priori, leaving 104 cases for internal development (architecture and schedule selection).

#### Annotation protocol

Public dataset annotations were accepted as released. External cohort liver and tumor masks were delineated by radiologists with consensus adjudication as necessary and exported in DICOM format (.dcm).

#### Preprocessing

Standard nnU-Net v2 preprocessing was applied: conversion to Hounsfield Units, intensity clipping to the range [−200, 250] HU, per-volume z-score normalization, isotropic resampling to 1.0 mm spacing (third-order spline for images; nearest neighbor for labels), and automatic foreground cropping. A 2D nnU-Net configuration was used throughout; axial slices were sampled with the default foreground oversampling probability (0.33). No additional custom preprocessing steps were introduced.

#### Data augmentation

The nnU-Net v2 default on-the-fly augmentation pipeline (spatial rotations, scaling, flips, elastic deformations, gamma/intensity adjustments, Gaussian noise/blur, low-resolution simulation) was applied identically across all architectures. No test-time augmentation was used.

## Class imbalance handling

Tumor voxels formed a small fraction of total volume. Mitigation relied on: (1) default foreground slice oversampling (0.33), (2) the nnU-Net composite Dice + cross-entropy loss with deep supervision, and (3) standard nnU-Net post-processing (largest liver component retention; removal of tiny isolated tumor components). No tumor-specific custom sampler or additional weighting was added.

## Baseline framework

All models were implemented on the nnU-Net v2 2D framework, sharing identical preprocessing, augmentation, loss formulation, optimizer schedule, inference procedure, and post-processing to ensure comparability.

## Modular architecture substitution

To ensure a fair and rigorous comparison, seven architectures were implemented within the identical framework: the 2D nnU-Net (our baseline), a standard U-Net, Attention U-Net, MultiRes U-Net, U-Net++ (NestedUNet), a vanilla TransUNet, and our proposed OF-TransUNet. Custom trainer subclasses replaced only the network instantiation while preserving all other pipeline components.

## Proposed output-focused TransUNet (OF-TransUNet)

**Fig. 1:**
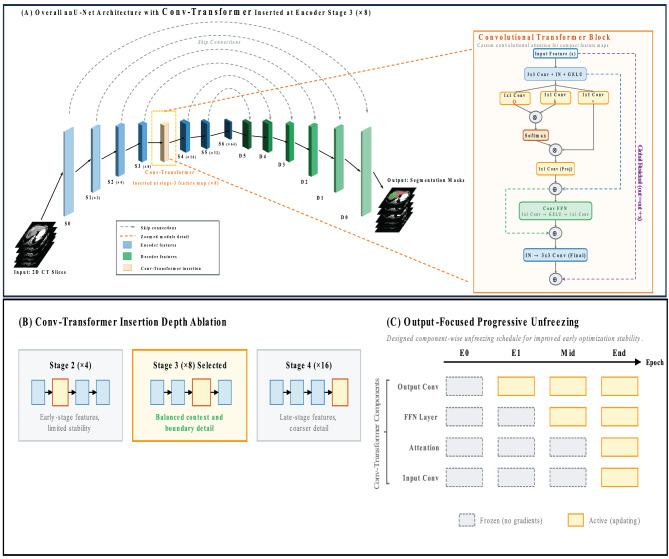
Output-focused TransUNet. (**A**) Overall nnU-Net encoder–decoder architecture with a single lightweight Conv-Transformer inserted after encoder stage 3 (×8). The inserted module preserves the original backbone and decoder while augmenting the stage-3 feature map with convolutional attention and residual feature refinement. (**B**) Insertion-depth ablation across stages 2, 3, and 4, with stage 3 providing the most favorable balance between contextual modeling and boundary detail on internal development data. (**C**) Designed output-focused progressive unfreezing schedule of the Conv-Transformer, in which output-proximal components are released before deeper internal components to improve early optimization stability during adaptation

We insert a single lightweight Conv-Transformer block after encoder stage 3 (downsampling × 8), chosen to balance retained spatial detail and emerging semantic abstraction. Unlike standard Vision Transformer modules that rely on tokenization and linear projections, our inserted block is convolutional throughout and is specifically tailored for compact medical feature maps. It uses convolutional QKV projection, attention projection, a convolutional feed-forward network (FFN), and residual output mapping, while preserving the original 2D nnU-Net backbone and decoder pathway. This restrained design captures long-range context with only modest overhead (+2.71 M trainable parameters relative to baseline). Pretrained weights were not used, to avoid confounding from heterogeneous pretraining corpora. Figure 1 illustrates (A) the overall nnU-Net architecture with a single Conv-Transformer inserted at stage 3, (B) the insertion-depth ablation across stages 2/3/4, and (C) the designed output-focused progressive unfreezing schedule used to guide staged adaptation of the Conv-Transformer components.

## Ablation design

### Transformer insertion depth

Insertion after encoder Stage 2 (×4), Stage 3 (×8), and Stage 4 (×16) was compared against the no-Transformer baseline (Fig. 1B). Stage 1 was excluded due to memory cost and limited anticipated global benefit.

### Fine-tuning strategies

Four training strategies were evaluated (the output-focused schedule is schematically illustrated in Fig. 1C):

1. **No Fine-tuning:** The inserted Conv-Transformer remained frozen throughout training; only the nnU-Net backbone and decoder were updated.
2. Full Fine-tuning: All Conv-Transformer parameter groups were trainable from the start of training.
3. Simple Progressive: After an initial stabilization period, the inserted Transformer was unfrozen sequentially using four predefined parameter groups in the implementation. In the implemented configuration, the Transformer remained frozen for epochs 0–69, after which one parameter group was released every 20 epochs in the default order [1–4], corresponding to release onsets at epochs 70, 90, 110, and 130.
4. Output-Focused (final): A refined staged unfreezing design in which output-proximal Transformer components were released before deeper internal components to improve early optimization stability during adaptation. In the final OF-TransUNet implementation, the inserted Transformer remained frozen during epochs 0–89. At epoch 90, the output-side mapping component ([“final”]) was released first, followed by [“final”, “conv2”, “conv3”] at epoch 110, [“qkv”, “proj”] at epoch 130, and full unfreezing at epoch 150 and thereafter. A short Transformer-specific learning-rate warmup was applied during epochs 90–94.

For the final OF-TransUNet schedule, the inserted Transformer was initialized in a frozen state during an early backbone stabilization phase, such that epochs 0–89 effectively trained the underlying nnU-Net backbone while keeping the Transformer branch inactive for parameter updates. At epoch 90, the output-side parameter group ([“final”]) was released and the optimizer was reconfigured to include the newly trainable parameters. A short Transformer-specific warmup was then applied during epochs 90–94. Additional parameter groups were released at epoch 110 ([“final”, “conv2”, “conv3”]) and epoch 130 ([“qkv”, “proj”]), followed by full unfreezing at epoch 150 and thereafter. Accordingly, the output-focused strategy should be interpreted as an explicitly implemented output-prioritized staged adaptation scheme.

### Training configuration

The baseline nnU-Net was trained for 200 epochs to provide a stable reference. All other candidate and ablation models were trained for at least 150 epochs (≥150) until convergence plateaus; extending beyond this did not yield material tumor Dice gains in internal monitoring. No early stopping was used.

Optimizer: stochastic gradient descent (initial learning rate 0.01; momentum 0.99; Nesterov = True) with polynomial decay (power 0.9) to zero over the allocated epochs. No global learning-rate warmup was used. For the final OF-TransUNet schedule only, a short Transformer-specific warmup was applied during epochs 90–94 after unfreezing began, with the Transformer learning rate increasing linearly toward its target branch-specific value.

Loss: nnU-Net composite Dice + cross-entropy with deep supervision (higher-resolution outputs assigned larger normalized weights; lowest-resolution head weight 0).

Mixed precision (AMP) was enabled. Random seed 42 was fixed. No ensembling or test-time augmentation was applied. In the standard nnU-Net training framework, epoch-wise validation Dice is logged directly during training, whereas training-side optimization is monitored primarily through training loss rather than epoch-wise training Dice. Accordingly, convergence visualization in this study includes both validation tumor Dice and training-loss trajectories over the shared main optimization window, with validation Dice serving as the primary Dice-based readout for direct architectural comparison.

Internal ablation results supported the need for staged adaptation. In the pooled internal benchmark summarized in Table 1, one immediate full fine-tuning configuration yielded complete failure (tumor Dice 0.0000). Additional repeated runs, including seed-controlled reruns, further indicated that immediate full fine-tuning showed unstable and poorly reproducible optimization behavior, ranging from near-zero collapse or low-level stagnation to only modest convergence. By contrast, both Simple Progressive and Output-Focused schedules yielded stable and reproducible training behavior in our development setting. Repeated NaN/Inf events were also observed in the final OF-TransUNet training log during the Transformer adaptation phase, indicating that staged unfreezing should be viewed as a practical stabilization strategy rather than a complete solution to optimization instability.

**Table 1:** Training strategy (internal validation)

Strategy	Tumor Dice (mean ± SD)	Δ vs Baseline (Abs/Rel %)	Δ vs Simple Progressive (Abs/Rel %)	Tumor IoU (mean ± SD)	Liver Dice (mean ± SD)	Δ Liver Dice (Rel %)	Collapsed?
Baseline (nnUNet)	0.3620 ± 0.3227	+0.0000/+0.00%	−0.1014/-21.88%	0.2736 ± 0.2723	0.8587 ± 0.1051	+0.00%	No
No Fine-tuning	0.0993 ± 0.1215	−0.2627/-72.58%	−0.3641/-78.58%	0.0570 ± 0.0758	0.5839 ± 0.1344	−32.00%	No
Full Fine-tuning	0.0000 ± 0.0000	−0.3620/-100.00%	−0.4634/-100.00%	0.0000 ± 0.0000	0.1494 ± 0.2011	−82.60%	Yes (collapsed)
Simple Progressive	0.4634 ± 0.3199	+0.1014/+28.01%	+0.0000/+0.00%	0.3591 ± 0.2817	0.8652 ± 0.1014	+0.75%	No
OF-TransUNet	**0.4655 ± 0.3069**	**+0.1035/+28.60%**	**+0.0021/+0.46%**	**0.3558 ± 0.2678**	**0.8638 ± 0.1005**	**+0.58%**	**No**

Notes: Δ vs Baseline uses Baseline (nnUNet) as reference; Δ vs Simple Progressive uses Simple Progressive strategy as reference. “Collapsed” denotes total failure (all tumor Dice ≈0). In the pooled internal benchmark, the evaluated immediate full fine-tuning configuration resulted in complete failure. Additional repeated runs and seed-controlled reruns indicated that immediate full fine-tuning showed unstable and poorly reproducible optimization behavior, whereas both Simple Progressive and Output-Focused schedules produced stable performance in our development setting. All values are descriptive only; no inferential statistics were performed on internal development data

### Inference and post-processing

Inference used sliding windows with 50% overlap and Gaussian weighting. Standard nnU-Net post-processing retained the largest liver component and removed small isolated tumor islands.

### Evaluation metrics

For each patient and each class (liver, tumor), we computed the following five metrics. Let *P* and *G* denote the predicted and ground-truth voxel sets, respectively, and let *V*_*P*_ and *V*_*G*_ denote their respective volumes. All Dice, IoU, VS, and HD95 metrics reported for the external cohort were computed at the per-patient level on whole-volume 3D voxel masks rather than slice-wise 2D masks.

**Dice Similarity Coefficient (DSC):**

1 $$SC = {{2\left| {P\mathop \cap \nolimits^ G} \right|} \over {}}\left( {\left| P \right| + \left| G \right|} \right)$$

**Intersection over Union (IoU):**

2 $$IoU = {{\left| {P\mathop \cap \nolimits^ G} \right|} \over {}}\left| {P\,\mathop \cup \ G} \right|$$

**Volume Similarity (VS):**

3 $$VS = 1 - {{\left| {{V_P} - {V_G}} \right|} \over {}}\left( {{V_P} + {V_G}} \right)$$

**Foreground Dice (DSC**_***FG***_), computed over the union of liver (subscript *L*) and tumor (subscript *T*) voxels:

4 $$DS{C_{FG}} = {{2\left| {\left( {{P_L}\mathop \cup {P_T}} \right)\mathop \cap \left( {{G_L}\mathop \cup {G_T}} \right)} \right|} \over {}}\left( {\left| {{P_L}\mathop \cup {P_T}} \right| + \left| {{G_L}\mathop \cup {G_T}} \right|} \right)$$

**95th Percentile Hausdorff Distance (HD95):** Let *d*(*p*,*G*) denote the shortest surface distance from point *p* to the surface of *G*. HD95 is defined as the 95th percentile of the set of all such distances computed bidirectionally:

5 $$HD95\left( {P,G} \right) = max\left( {perc95\left\{ {d\left( {p,G} \right):p \in \partial P} \right\}, perc95\left\{ {d\left( {g,P} \right):g \in \partial G} \right\}} \right)$$

where ∂*P* and ∂*G* denote the surface voxel sets of *P* and *G*, respectively. Infinite or undefined surface distances arising from empty predictions were excluded from HD95 aggregation; the effective per-class sample size after exclusion is reported in table footnotes.

Metrics were computed per patient; aggregate results are reported as mean ± standard deviation. Handling of invalid distances: If a model produced an empty prediction for a given class (or a class with no ground-truth voxels), any resulting infinite or undefined surface distance was excluded from the HD95 aggregation for that class.

## Computational efficiency assessment

To substantiate the “minimal” characterization, we quantified computational overhead only for the direct architectural contrast of interest: the standard 2D nnU-Net baseline versus the proposed OF-TransUNet. Other comparator architectures (e.g., U-Net++, MultiResUNet, TransUNet) were not profiled because the study’s efficiency question centers on whether a single mid-level Transformer insertion materially alters the baseline deployment profile.Metrics included total trainable parameters (Millions), manually computed floating-point operations (FLOPs, GigaFLOPs) for a single 512 × 512 2D input, average per-slice inference latency (milliseconds), frames per second (FPS = 1000/latency), and peak inference GPU memory usage. Latency was averaged over repeated forward passes (≥100 iterations after 10 warm-up runs) on a single NVIDIA RTX 4080 (16 GB), with automatic mixed precision enabled uniformly across both models. Peak memory was obtained via monitoring (torch.cuda.max_memory_allocated) after stabilization. No kernel-level or graph-level inference optimizations (e.g., TensorRT, operator fusion) were applied. All measurements reflect the 2D configuration used in the principal experiments and are intended for relative (not absolute, cross-hardware) interpretation. Minor latency differences (<1 ms) are interpreted cautiously and may reflect scheduling noise or implementation-level kernel variation rather than intrinsic architectural acceleration.

### Primary endpoint

The pre-specified primary endpoint was the per-patient tumor Dice similarity coefficient (DSC) difference between OF-TransUNet and the nnU-Net baseline on the external validation cohort (*n* = 42). Per-patient Dice (mean ± SD) was computed on whole-volume 3D voxel masks for each model. Normality of the paired Dice differences was assessed using the Shapiro–Wilk test. Because the paired differences deviated from normality, the two-sided Wilcoxon signed-rank test was retained as the pre-specified primary inferential analysis. In addition, the mean paired difference and its 95% confidence interval were reported, and a paired t-test was included as a supplementary sensitivity analysis of the mean paired difference. The reported 95% CI corresponds to the mean paired Dice difference rather than the median paired difference.

### Secondary Lesion-Level Analysis: medium-sized (10–50 mm) lesions

A pre-defined secondary lesion-level analysis focused on medium-sized tumors (10–50 mm), justified by (i) their high aggregate prevalence in large-scale lesion mining studies [34], and (ii) evidence that state-of-the-art liver tumor segmentation performance increases with lesion size but has not saturated in this diameter range [35]. This analysis was restricted to ground-truth lesions within 10–50 mm maximum in-plane diameter.

### Lesion detection criteria

A lesion was considered detected (true positive) if the volumetric overlap ratio, defined as:

6 $$overlap\,Ratio = {{\left| {P\mathop \cap \nolimits^ {G_{lesion}}} \right|} \over {}}\left| {{G_{lesion}}} \right| \ge 0.10$$

where G_lesion denotes the voxel set of a single ground-truth lesion component. Lesions below this threshold were classified as false negatives. The 10% threshold was pre-specified for the primary lesion-level secondary analysis.

### Statistical Methods (lesion-level secondary analysis)

1. Detection Rate: For medium-sized lesions, paired detection outcomes (detected vs missed) between each candidate model and the baseline nnU-Net were compared using McNemar’s exact test.
2. GEE Model: A Generalized Estimating Equation (GEE) logistic regression assessed the association between model type and lesion-level detection probability while accounting for intra-patient clustering. The outcome was binary detection (yes/no). Covariates included model indicator variables (nnU-Net as reference) and lesion diameter (continuous, mm). An exchangeable working correlation structure was specified, and robust (sandwich) standard errors were used. The analysis included all medium-sized lesions (*n* = 84) and was implemented in Python using the statsmodels library. For concise reporting in the main text, we highlight the adjusted odds ratio (OR) comparing OF-TransUNet to nnU-Net; ORs for other models are tabulated.
3. Net Detection Gain: Defined as (lesions detected by the model but missed by nnU-Net) − (lesions missed by the model but detected by nnU-Net).
4. Multiplicity: No multiplicity adjustment was applied for multiple model contrasts in this exploratory secondary analysis; *p*-values should be interpreted accordingly.
5. Confidence Intervals: For the primary endpoint, the 95% confidence interval reported in the main text corresponds to the mean paired difference. For lesion-level detection rates, 95% CIs were computed using the Wilson score method. For odds ratios derived from logistic and GEE models, 95% CIs were based on model-based robust standard errors.

As a post hoc robustness check, additional sensitivity analyses were performed using alternative lesion-detection overlap thresholds of 5 and 15%, while retaining the same medium-lesion subset, paired McNemar framework, and diameter-adjusted regression models. These analyses were not pre-specified and are therefore interpreted as supportive rather than confirmatory. Among model-wise secondary comparisons, only OF-TransUNet versus nnU-Net was pre-specified for confirmatory interpretation; all other model-wise contrasts were exploratory and unadjusted for multiplicity.

## Internal development data: descriptive use only

All internal development experiments (public HCC-TACE dataset, *n* = 104) were conducted strictly for descriptive architecture and training strategy selection. No hypothesis testing or multiplicity-adjusted inference was performed on internal metrics. The pre-specified primary confirmatory endpoint (per-patient tumor Dice improvement) and all inferential statistics were reserved exclusively for the independent external validation cohort (*n* = 42).

## Supplementary LiTS benchmark design

To address the editorial request for public-benchmark contextual validation, we performed a supplementary controlled benchmark on the Liver Tumor Segmentation Benchmark (LiTS) dataset. Because the official LiTS hidden test set does not provide publicly released ground-truth segmentations for local quantitative evaluation, this supplementary analysis was necessarily restricted to the 131 publicly available annotated LiTS cases rather than the 70 hidden test cases.

Due to time and computational resource constraints, the supplementary LiTS benchmark was conducted on a single pre-fixed standard validation split (fold_0; *n* = 27 validation cases) rather than a full multi-fold rerun across all candidate models. This analysis was therefore designed as supportive contextual evidence rather than as a definitive LiTS-wide performance estimate.

All supplementary LiTS experiments were implemented within the identical 2D nnU-Net v2 pipeline used throughout the main study, sharing the same preprocessing, augmentation, loss formulation, optimizer schedule, inference procedure, and post-processing settings. Only the network instantiation differed. The evaluated models were the standardized 2D nnU-Net baseline, OF-TransUNet, a vanilla Transformer-augmented comparator implemented within the same framework (CustomTransUNet), and MultiResUNet.

We deliberately prioritized a strictly controlled within-pipeline comparison rather than aggregating raw published numbers as if they were directly comparable, because LiTS results reported in the literature are obtained under heterogeneous split strategies, dimensionalities, preprocessing pipelines, and inference configurations. Accordingly, the supplementary LiTS benchmark and the descriptive literature-based contextual table are interpreted separately in the present manuscript.

## Ethics

The study was approved by the institutional ethics committee with waiver of informed consent due to its retrospective, de-identified design, consistent with the Declaration of Helsinki. Use of the public HCC-TACE dataset complied with its license.

## Code availability

A public repository for OF-TransUNet has been created at https://github.com/callhe-ai/OF-TransUNet. During peer review, the repository includes the core trainer implementation (`nnUNetTrainerTransUNet.py`), repository-level documentation, dependency information, and supplementary implementation notes describing the staged Transformer unfreezing schedules. The core implementation is therefore already publicly available for transparency during peer review, while complete training and inference scripts, evaluation scripts, pretrained weights, and full reproducibility documentation will be finalized and expanded upon manuscript acceptance. The repository will also include step-by-step instructions for reproducing the principal quantitative results reported in Tables 2–3, 4.

**Table 2:** Comprehensive performance on external validation cohort (*n* = 42 patients)

Model	Liver Segmentation				Tumor Segmentation				Wilcoxon **p-value**^**1**^
	Dice	IoU	VS	HD95 (mm)	Dice	IoU	VS	HD95 (mm)	
nnU-Net (Baseline)	0.767 ± 0.116	0.635 ± 0.137	0.910 ± 0.087	19.222 ± 23.872	0.240 ± 0.243	0.160 ± 0.175	0.550 ± 0.283	96.776 ± 88.131	—
**OF-TransUNet (Ours)**	0.758 ± 0.120	0.624 ± 0.144	0.890 ± 0.110	17.218 ± 22.883	0.279 ± 0.257	0.190 ± 0.190	0.578 ± 0.332	104.353 ± 74.730	0.0553
TransUNet	0.734 ± 0.141	0.597 ± 0.159	0.887 ± 0.121	20.297 ± 26.824	0.256 ± 0.253	0.173 ± 0.186	0.587 ± 0.313	103.480 ± 85.124	0.5966
MultiResUNet	0.742 ± 0.122	0.603 ± 0.143	0.888 ± 0.093	33.004 ± 30.937	0.262 ± 0.250	0.176 ± 0.183	0.619 ± 0.305	111.205 ± 82.316	0.1574
AttUNet	0.717 ± 0.131	0.573 ± 0.149	0.881 ± 0.120	21.462 ± 25.570	0.218 ± 0.241	0.146 ± 0.177	0.519 ± 0.315	98.816 ± 84.284	0.0841
CustomUNet	0.764 ± 0.113	0.630 ± 0.133	0.907 ± 0.079	27.262 ± 28.004	0.244 ± 0.251	0.164 ± 0.184	0.589 ± 0.307	102.047 ± 80.772	0.8186
NestedUNet	0.758 ± 0.130	0.626 ± 0.151	0.897 ± 0.104	19.394 ± 26.189	0.216 ± 0.239	0.143 ± 0.172	0.575 ± 0.291	103.218 ± 84.029	0.0644

**Notes**

^1^ Two-sided Wilcoxon signed-rank test comparing each model’s per-patient tumor Dice with the nnU-Net baseline across the same 42 patients. OF-TransUNet vs nnU-Net was the only pre-specified confirmatory comparison; all other model comparisons are exploratory. No multiplicity adjustment was applied

Dice values are per-patient 3D volumetric Dice computed on whole-volume voxel masks rather than slice-wise or lesion-level Dice

*Primary endpoint (re-verified):* OF-TransUNet tumor Dice improvement = +0.0388 (95% CI for mean paired difference: 0.0029 to 0.0748; Wilcoxon *p* = 0.0553; paired t-test *p* = 0.0347; Shapiro–Wilk p for paired differences = 0.0007799)

Because the paired tumor Dice differences deviated from normality, the Wilcoxon signed-rank test was retained as the pre-specified primary inferential analysis; the paired t-test is provided as a supplementary sensitivity analysis only.

**Abbreviations:** Dice, Dice similarity coefficient; IoU, Intersection over Union; VS, Volumetric Similarity; HD95, 95th percentile Hausdorff Distance

**Table 3:** Performance comparison on medium-sized lesions (10–50 mm, *n* = 84)

Model	Detection Rate	vs. nnU-Net (Abs. Diff.)	McNemar’s Test (p-value)	Adjusted Odds Ratio **(95% CI)**^**1**^	GEE Model (p-value)	**Net Detection Gain**^**2**^
nnU-Net (Baseline)	0.190	—	—	1.00 (Reference)	—	—
**OF-TransUNet (Ours)**	**0.286**	**+0.095**	0.021*	**1.97 (1.15–3.35)**	0.013*	**+6 (11 vs 5)**
TransUNet	**0.298**	**+0.107**	0.035*	1.94 (0.98–3.85)	0.058	+8 (14 vs 6)
MultiResUNet	**0.286**	**+0.095**	0.039*	**1.82 (1.02–3.22)**	0.041*	+11 (15 vs 4)
AttUNet	0.262	+0.071	0.180	**1.58 (1.01–2.49)**	0.046*	+4 (12 vs 8)
CustomUNet	0.250	+0.060	0.267	1.47 (0.85–2.57)	0.170	+6 (12 vs 6)
NestedUNet	0.143	−0.048	0.219	0.69 (0.46–1.04)	0.074	−7 (2 vs 9)

**Notes**

1. Odds ratio from the Generalized Estimating Equations (GEE) model, adjusted for lesion diameter

2. Net gain calculated as (lesions detected by model but missed by nnU-Net) − (lesions missed by model but detected by nnU-Net)

All *p*-values are two-sided. McNemar’s test uses exact paired comparisons. OF-TransUNet vs nnU-Net was the only pre-specified confirmatory model comparison; all other model-wise contrasts are exploratory and unadjusted for multiplicity. The primary lesion-level definition used the pre-specified 10% overlap threshold; post hoc sensitivity analyses using 5 and 15% thresholds are reported in Supplementary Table S1

**Table 4:** Computational efficiency comparison (2D configuration; single 512 × 512 slice)

Model	Params (M)	Δ vs Baseline (%)	FLOPs (G)	Δ vs Baseline (%)	Avg Latency (ms)	FPS	Peak Memory (MB)
nnU-Net (Baseline)	**32.42**	—	**22.69**	—	**10.24**	**97.65**	**44.35**
OF-TransUNet	35.13	+8.4	26.84	+18.3	**9.55**	**104.70**	45.47

**Notes**

FLOPs manually computed for a single 2D 512 × 512 forward pass including all convolutions, normalization, and attention operations

Latency averaged over ≥ 100 runs post warm-up on NVIDIA RTX 4080 (mixed precision enabled, CUDA 11.8, PyTorch 1.13)

Minor latency decrease (<1 ms) is interpreted as within measurement variability rather than intrinsic acceleration

Peak Memory refers to maximum GPU memory allocated during single-slice inference (batch size = 1).

FPS = 1000/(latency in ms), representing theoretical maximum throughput for continuous single-slice processing.

Δ vs Baseline = (Model − Baseline)/Baseline × 100%.

Bold indicates optimal values: minimum latency and maximum FPS demonstrate computational efficiency despite modest parameter increase.

## Data availability

The HCC-TACE dataset is publicly accessible [4]. The external clinical dataset is not publicly released due to institutional data-sharing restrictions. De-identified derived results, such as per-patient segmentation metrics and lesion-level detection summary tables, may be shared upon reasonable request, subject to institutional approval and applicable data-sharing restrictions. Requests should be directed to the corresponding author, Xiaoping Pan (http://pxp74@sina.com).

## Qualitative visualization

Representative examples (internal and external cohorts) highlighting small lesions and enhancement variability were selected under a uniform color scheme (liver: red; tumor: green fill with green boundary) for illustrative purposes only.

## Results

In small target segmentation tasks, such as those involving liver tumors, class imbalance and high inter-lesion variability present significant challenges. Our results are presented by first detailing the comprehensive multi-model benchmark on the primary external validation cohort, followed by supporting evidence from our internal development and ablation studies.

### Primary endpoint: external per-patient tumor Dice

The re-verified primary endpoint analysis on the external cohort (*n* = 42) showed that OF-TransUNet yielded a higher per-patient tumor Dice than the nnU-Net baseline (0.2788 ± 0.2575 vs 0.2400 ± 0.2426), corresponding to a mean paired difference of +0.0388 (95% CI for the mean paired difference, 0.0029 to 0.0748). The paired Dice differences deviated from normality (Shapiro–Wilk *p* = 0.00078). Accordingly, the pre-specified two-sided Wilcoxon signed-rank test was retained as the primary inferential analysis and did not reach the conventional 0.05 threshold (*p* = 0.0553). For completeness, a supplementary paired t-test on the same paired data yielded nominal significance (*p* = 0.0347; Cohen’s dz = 0.337), supporting the direction of effect but not replacing the pre-specified non-parametric inference. Liver segmentation metrics remained broadly comparable across models (Table 2), suggesting that the observed directional gain was concentrated in the tumor class.

To contextualize this result within the broader benchmark, Table 2 summarizes all seven architectures evaluated on the external validation cohort (*n* = 42). All comparator models were evaluated within the same controlled nnU-Net v2 pipeline, sharing identical preprocessing, augmentation, loss formulation, inference, and post-processing settings; only the network instantiation differed. Statistical comparisons in Table 2 were based on two-sided Wilcoxon signed-rank tests for paired per-patient tumor Dice differences relative to the nnU-Net baseline. OF-TransUNet versus nnU-Net was the only pre-specified confirmatory comparison; all other model contrasts were exploratory and were not adjusted for multiplicity.

As shown in Table 2, OF-TransUNet achieved the highest mean per-patient tumor Dice among the evaluated models (0.2788 ± 0.2575). However, under the pre-specified Wilcoxon analysis, this paired improvement was borderline and did not reach the conventional 0.05 threshold. Tumor HD95 was numerically higher for OF-TransUNet than for nnU-Net (104.353 ± 74.730 vs 96.776 ± 88.131), suggesting a possible recall–boundary trade-off. Because the current evidence for this interpretation is limited to aggregate metrics and a small post-hoc qualitative review, it should be regarded as cautious and hypothesis-generating rather than definitive. As a descriptive post hoc robustness summary, we further examined paired case-level changes in tumor HD95 among cases with valid tumor HD95 in both nnU-Net and OF-TransUNet (*n* = 39). Relative to nnU-Net, OF-TransUNet showed worsened tumor HD95 in 25/39 cases (64.1%), including increases greater than 5 mm in 18/39 cases (46.2%) and greater than 10 mm in 11/39 cases (28.2%). Conversely, tumor HD95 improved in 14/39 cases (35.9%), including decreases greater than 5 mm in 10/39 cases (25.6%) and greater than 10 mm in 8/39 cases (20.5%). These descriptive findings support that the aggregate HD95 increase was heterogeneous across cases rather than uniformly worsened in all patients. Overall, these findings support a modest patient-level tumor segmentation gain in the proposed model, while indicating that the strongest statistically supported benefit in this dataset was observed in the pre-defined medium-lesion detection analysis described below.

As shown in Table 2, OF-TransUNet achieved the highest mean per-patient tumor Dice among the evaluated models, while liver segmentation metrics remained within a broadly comparable range across architectures. Under the pre-specified Wilcoxon analysis, however, the OF-TransUNet versus nnU-Net paired tumor Dice comparison was borderline rather than conventionally significant. Tumor HD95 was numerically higher for OF-TransUNet, which is compatible with a possible recall–boundary trade-off, although the present study does not provide sufficient evidence for a definitive mechanistic explanation. Taken together, these results support a modest and directionally favorable patient-level tumor segmentation gain under a restrained architectural modification, while the clearest statistically supported advantage in this dataset was seen in the pre-defined medium-lesion detection analysis.

### Secondary Lesion-Level Analysis: medium-sized (10–50 mm) lesions

This pre-defined secondary lesion-level analysis examined 84 medium-sized (10–50 mm) tumors in the external cohort, a diameter range selected for its high prevalence and intermediate difficulty. Detailed detection metrics, paired McNemar comparisons versus the nnU-Net baseline, net detection gain, and GEE-adjusted odds ratios are summarized in Table 3.

*OF-TransUNet increased the medium-lesion detection rate, defined by* Eq. 6*using the pre-specified 10% volumetric overlap threshold, from 0.190 to 0.286 (absolute +0.095; relative +50.0%).* This improvement was statistically significant by McNemar’s exact test (*p* = 0.021, unadjusted). MultiResUNet (*p* = 0.039) and TransUNet (*p* = 0.035) also showed nominal (unadjusted) detection-rate improvements over nnU-Net. Other variants (e.g., NestedUNet) failed to exceed baseline performance.

Accounting for patient-level clustering and continuous lesion diameter in a GEE logistic model (exchangeable correlation; robust standard errors), OF-TransUNet achieved an adjusted odds ratio (OR) of 1.97 (95% CI 1.15–3.35; *p* = 0.013) relative to nnU-Net. AttUNet and MultiResUNet likewise produced adjusted ORs with confidence intervals excluding 1.0 (Table 3). These *p*-values are exploratory; no multiplicity adjustment was applied across multiple model contrasts.

Post hoc sensitivity analyses using alternative overlap thresholds showed that the lesion-level advantage of OF-TransUNet over nnU-Net was directionally robust. At a lenient 5% threshold, detection increased from 0.226 to 0.286 (absolute +0.060), although the paired difference did not reach conventional significance (McNemar exact *p* = 0.227; GEE OR 1.51, 95% CI 0.92–2.46; *p* = 0.101). At the pre-specified 10% threshold, detection increased from 0.190 to 0.286 (absolute +0.095; McNemar exact *p* = 0.021; GEE OR 1.97, 95% CI 1.15–3.35; *p* = 0.013). At the stricter 15% threshold, detection increased from 0.155 to 0.262 (absolute +0.107; McNemar exact *p* = 0.035; GEE OR 2.20, 95% CI 1.16–4.16; *p* = 0.016). This pattern suggests that the observed benefit was not solely driven by minimal overlap events near the detection boundary (Supplementary Table S1).

**Fig. 2:**
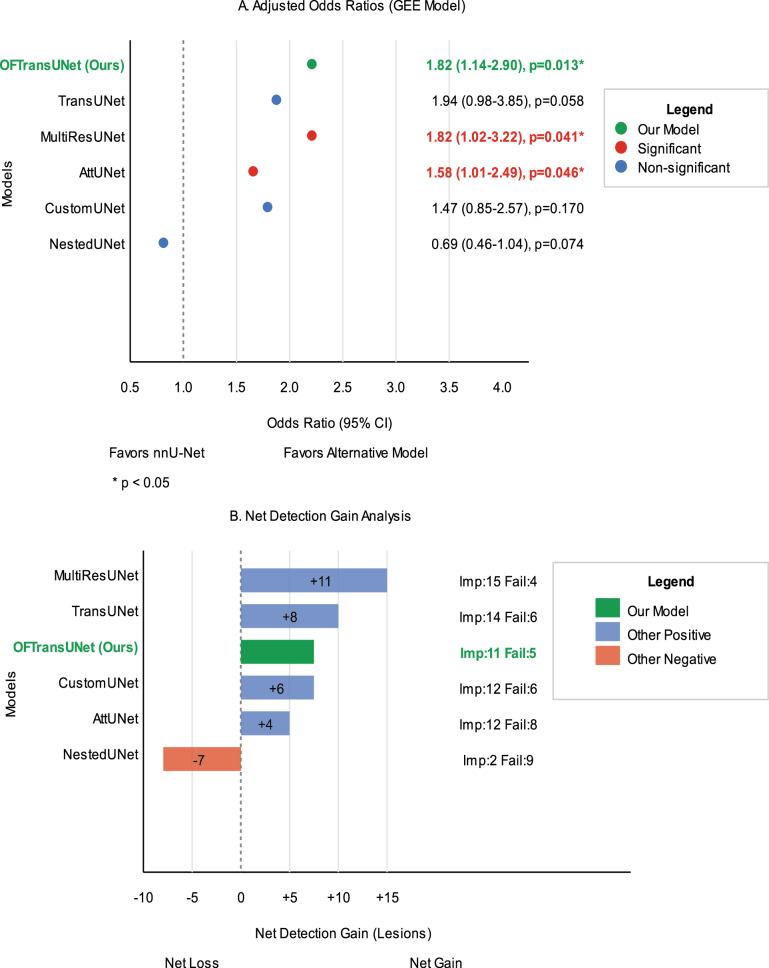
Forest plot of adjusted odds ratios and net detection gain for medium-sized lesions. Notes: panel a shows adjusted odds ratios from the GEE model with 95% confidence intervals. Panel B shows net detection gain = (lesions detected by the model but missed by nnU-Net) − (lesions missed by the model but detected by nnU-Net). *Nominal *p* < 0.05 versus the nnU-Net baseline; except for OF-TransUNet vs nnU-Net, all model-wise comparisons are exploratory and unadjusted for multiplicity

**Fig. 3:**
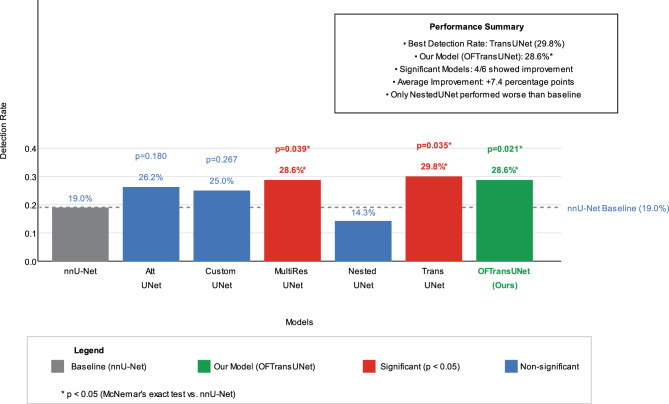
Comparison of detection rates for medium-sized (10–50 mm) lesions across evaluated models

In summary, this pre-defined secondary medium-lesion analysis shows that OF-TransUNet delivers a statistically supported improvement in lesion detection probability under a controlled architectural change, complementing the numerically favorable but statistically borderline patient-level Dice result reported in Sect. Primary endpoint: external per-patient tumor Dice. Figure 2A(forest plot) visualizes adjusted ORs, with OF-TransUNet, MultiResUNet, and AttUNet exhibiting confidence intervals entirely above unity. Figure 2Bpresents net detection gain, defined as (lesions detected by the model but missed by nnU-Net) minus (lesions missed by the model but detected by nnU-Net). MultiResUNet achieved the largest net gain (+11), while OF-TransUNet produced a positive net gain of + 6 (11 additional lesions detected; 5 missed that baseline detected), reinforcing a clinically relevant benefit without a large false-substitution penalty. Figure 3 (bar chart) contrasts raw medium-lesion detection rates across all models, illustrating a performance tier comprising TransUNet, OF-TransUNet, and MultiResUNet above the nnU-Net baseline, with NestedUNet underperforming.

### Computational efficiency (baseline vs single-block integration)

Table 4 summarizes the computational profile of the proposed single-block Transformer insertion relative to the nnU-Net baseline. OF-TransUNet increased parameter count from 32.42 M to 35.13 M (+8.4%) and FLOPs from 22.69 G to 26.84 G (+18.3%) for a 512 × 512 slice. Despite this moderate arithmetic overhead, average per-slice inference latency was not penalized (10.24 ms vs 9.55 ms; difference within typical measurement variability), resulting in a comparable effective throughput (97.65 vs 104.70 FPS). Peak inference memory usage increased by only ~1.1 MB (44.35 vs 45.47 MB). These findings empirically support that the performance gains described in Sect. Primary endpoint: external per-patient tumor dice were achieved without a substantial runtime or memory burden.

The efficiency characterization (Sect. “Computational efficiency (baseline vs single-block integration)”) confirms that subsequent architectural and training strategy ablations (Sect.“Internal development and ablation studies”– “Convergence behavior during the main optimization phase”) can be interpreted without confounding from large resource shifts.

### Supplementary LiTS benchmark and contextual comparison

To further contextualize the proposed architecture on a widely used public benchmark, we conducted a supplementary controlled experiment on the public LiTS dataset using the 131 publicly annotated cases only. Because the official LiTS hidden test labels are not publicly available for local quantitative evaluation, this supplementary benchmark was not intended to reproduce the official leaderboard protocol. Instead, it was designed to provide architecture-level contextual evidence under identical pipeline conditions.

On the pre-fixed fold_0 validation set (*n* = 27), OF-TransUNet achieved a higher per-patient tumor Dice than the standardized 2D nnU-Net baseline (0.5325 ± 0.3049 vs 0.5012 ± 0.3265), corresponding to a mean paired difference of +0.0313 (95% CI for the mean paired difference: −0.0122 to 0.0749). Because the paired differences were non-normal (Shapiro–Wilk *p* = 0.0307), the Wilcoxon signed-rank test was used and reached the conventional 0.05 threshold in this supplementary analysis (*p* = 0.0339), with 18 of 27 cases improved, 8 worsened, and 1 tied. The supplementary paired t-test did not reach significance (*p* = 0.1514), consistent with the small effect size (Cohen’s dz = 0.284).

Under the same controlled pipeline, the vanilla Transformer-augmented comparator (CustomTransUNet) showed a smaller, non-significant improvement over baseline (mean paired difference +0.0213; Wilcoxon *p* = 0.0875), while MultiResUNet showed no advantage over baseline (mean paired difference −0.0036; Wilcoxon *p* = 0.7164). These supplementary results therefore place OF-TransUNet as the most favorable of the evaluated lightweight comparators within this restricted fold_0 setting.

We emphasize that this LiTS analysis was performed on a single pre-fixed validation fold due to time and computational resource constraints, and should therefore be interpreted as supplementary contextual evidence rather than a definitive LiTS-wide performance estimate. The primary confirmatory analyses of the present study remain the pre-specified external validation results reported in Sects. Primary Endpoint: External Per-Patient Tumor Dice and Secondary lesion-level analysis: medium-sized (10–50 mm) lesions. Nevertheless, the direction of the supplementary LiTS finding is consistent with the external cohort results and provides additional support for the generalizability of the proposed approach under a controlled pipeline.

For descriptive context only, representative published LiTS tumor-segmentation results obtained under heterogeneous settings are summarized in Supplementary Table S3 [35–39]; these values are not interpreted as direct head-to-head comparisons.

### Internal development and ablation studies

#### Note on internal data interpretation

All internal development results reported in this section (Tables 5–6) are descriptive and were used solely for architecture and schedule selection. No hypothesis testing was performed; metrics serve to document the empirical basis for selecting OF-TransUNet for external validation. Primary confirmatory inference is reported in Sects. Primary Endpoint: External Per-Patient Tumor Dice and Secondary lesion-level analysis: medium-sized (10–50 mm) lesions. on the independent external cohort.

**Table 5:** Pooled internal development performance of candidate architectures (*n* = 104)

Model	Mean Dice (mean ± SD, pooled per-patient)			Δrel% vs Baseline	
	Liver	Tumor	**Foreground**^**a**^	Tumor	Foreground
nnU-Net (Baseline)	**0.8587 ± 0.1051**	**0.3620 ± 0.3227**	**0.8357 ± 0.1137**	—	—
OF-TransUNet	**0.8638 ± 0.1005**	**0.4655 ± 0.3069**	**0.8460 ± 0.1019**	+28.6	+1.2
MultiRes U-Net	0.8577 ± 0.1048	0.3719 ± 0.3198	0.8350 ± 0.1137	+2.7	−0.1
Vanilla TransUNet	0.8585 ± 0.1060	0.3730 ± 0.3218	0.8366 ± 0.1116	+3.0	+0.1
U-Net	0.8569 ± 0.1039	0.3694 ± 0.3224	0.8344 ± 0.1110	+2.0	−0.2
U-Net++	0.8571 ± 0.1056	0.3686 ± 0.3198	0.8348 ± 0.1120	+1.8	−0.1
Attention U-Net	0.8565 ± 0.1059	0.3687 ± 0.3200	0.8344 ± 0.1124	+1.9	−0.2

**Notes**

a. Foreground Dice computed over the union of liver and tumor voxels.

Values are pooled per-patient means ± standard deviations across 104 internal validation cases aggregated over five mutually exclusive folds (not SD of fold means).

Δrel% = (Model − Baseline)/Baseline × 100%, reported for tumor and foreground Dice only

Internal metrics are descriptive and used solely for architecture selection; no hypothesis testing or multiplicity-adjusted inference was performed.

Primary confirmatory inference (tumor Dice improvement) is reported on the independent external cohort (Table 2)

Bold indicates the highest mean value in each Dice column

Case count note: *n* = 104 reflects pooled validation cases. One case (HCC*_089) was excluded a priori due to missing/invalid annotation (see Section 2.3)*

To arrive at our final proposed architecture and training strategy, we conducted extensive internal experiments on the development dataset (*n* = 104).

First, an architectural benchmark was performed. As shown in Table 5, the OF-TransUNet achieved a tumor Dice of 0.4655, a substantial relative improvement of 28.6% over the nnU-Net baseline (0.3620). This result guided our decision to select OF-TransUNet for the primary external validation.

Furthermore, to validate our specific design choices, we performed ablation studies. As detailed in Tables 6 and 1, inserting the Transformer block at Stage 3 and employing a progressive, output-focused unfreezing strategy were both important to achieving optimal performance. Intermediate insertion (Stage 3) yielded the highest tumor Dice (0.4655), while one pooled immediate full fine-tuning run in the internal benchmark resulted in complete failure (Tumor Dice of 0.0000). Additional repeated runs, including seed-controlled reruns, showed that immediate full fine-tuning was not reliably trainable in this setting, with outcomes ranging from collapse or severe degradation to only modest convergence. These observations supported the use of staged adaptation rather than immediate full fine-tuning.

**Table 6:** Transformer insertion position (internal validation)

Method	Tumor Dice (mean ± SD)	Δ vs Baseline (Abs/Rel %)	Tumor IoU (mean ± SD)	Liver Dice (mean ± SD)	Δ Liver Dice (Rel %)
Baseline (nnUNet)	0.3620 ± 0.3227	+0.0000/+0.00%	0.2736 ± 0.2723	0.8587 ± 0.1051	+0.00%
Insert Stage 2	0.3624 ± 0.3097	+0.0004/+0.11%	0.2695 ± 0.2603	0.8331 ± 0.1214	−2.99%
**Insert Stage 3**	**0.4655 ± 0.3069**	**+0.1035/+28.60%**	**0.3558 ± 0.2678**	**0.8638 ± 0.1005**	**+0.58%**
Insert Stage 4	0.4528 ± 0.3115	+0.0908/+25.09%	0.3461 ± 0.2713	0.8530 ± 0.1102	−0.67%

Notes: Metrics are pooled per-patient across internal validation folds. Δ = absolute/relative change vs Baseline (nnUNet). Best tumor Dice row highlighted. Liver Dice is shown as a stability descriptor. All SD values use sample SD (ddof = 1). No hypothesis testing (descriptive only)

To further assess whether this behavior reflected a single anomalous run, we additionally examined repeated immediate full fine-tuning runs, including seed-controlled reruns. The resulting trajectories remained highly variable: some runs collapsed to near-zero validation pseudo Dice or degraded to persistent low-level performance, whereas others remained trainable but reached only modest plateaus. These observations indicate poor reproducibility and unstable optimization under immediate full fine-tuning in our setting.

### Convergence behavior during the main optimization phase

**Fig. 4:**
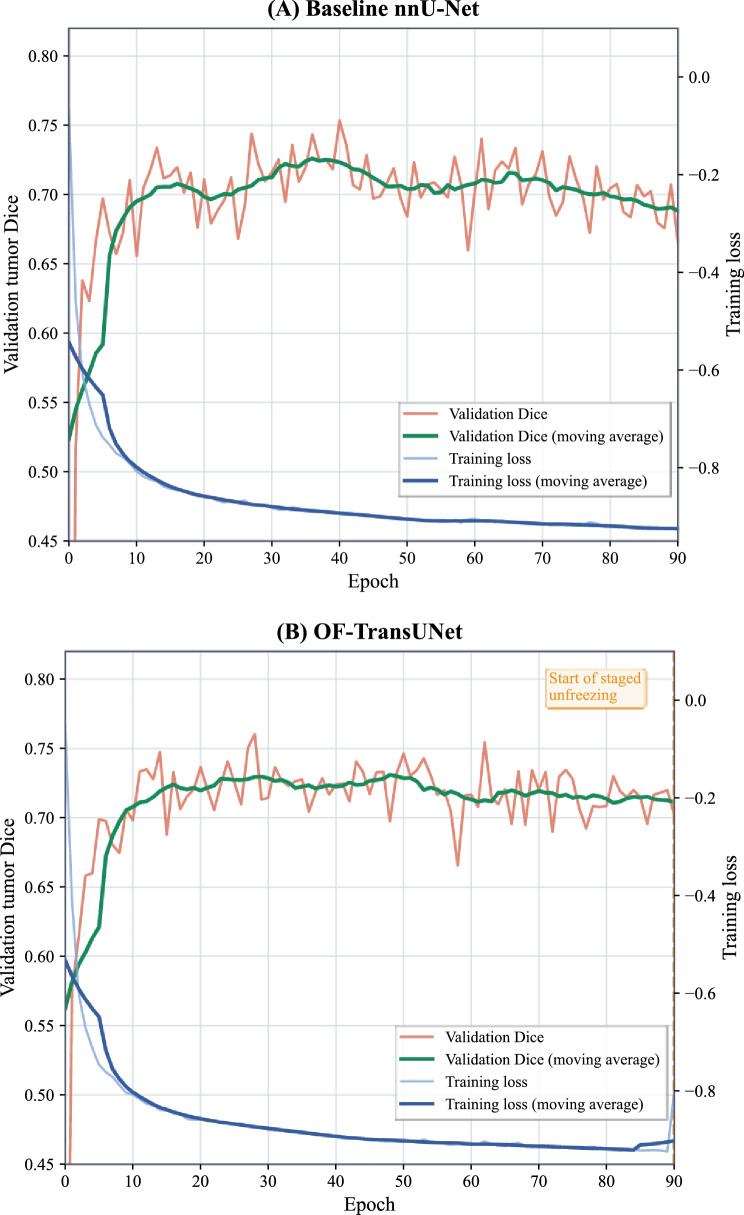
Convergence during the shared main optimization window on the internal development set. (**A**) baseline 2D nnU-Net. (**B**) proposed OF-TransUNet. Each panel shows tumor validation Dice and training loss recorded during training together with 11-epoch moving-average trend lines over the shared main optimization window (epochs 0–90). For OF-TransUNet, the dashed line marks epoch 90, when staged Conv-Transformer adaptation begins. The first released output-side component group corresponded to [“final”]

Figure 4 summarizes convergence behavior during the shared main optimization window (epochs 0–90) on the internal development set for the baseline 2D nnU-Net and the proposed OF-TransUNet. This interval was selected to provide a like-for-like comparison of the principal optimization phase shared by both models before staged Conv-Transformer unfreezing begins in OF-TransUNet.

Each panel displays both validation tumor Dice and training loss, together with 11-epoch moving-average trend lines. Across this shared window, both models showed stable optimization behavior, with OF-TransUNet reaching a slightly higher validation Dice plateau while maintaining a training-loss trajectory broadly comparable to the baseline. In the standard nnU-Net training framework, training-side convergence is monitored through loss rather than epoch-wise training Dice; therefore, the combined presentation of validation Dice and training loss provides a more complete view of optimization behavior while preserving validation Dice as the primary Dice-based comparison metric. For OF-TransUNet, the dashed line at epoch 90 marks the onset of staged Conv-Transformer adaptation.

### Qualitative visualization

**Fig. 5:**
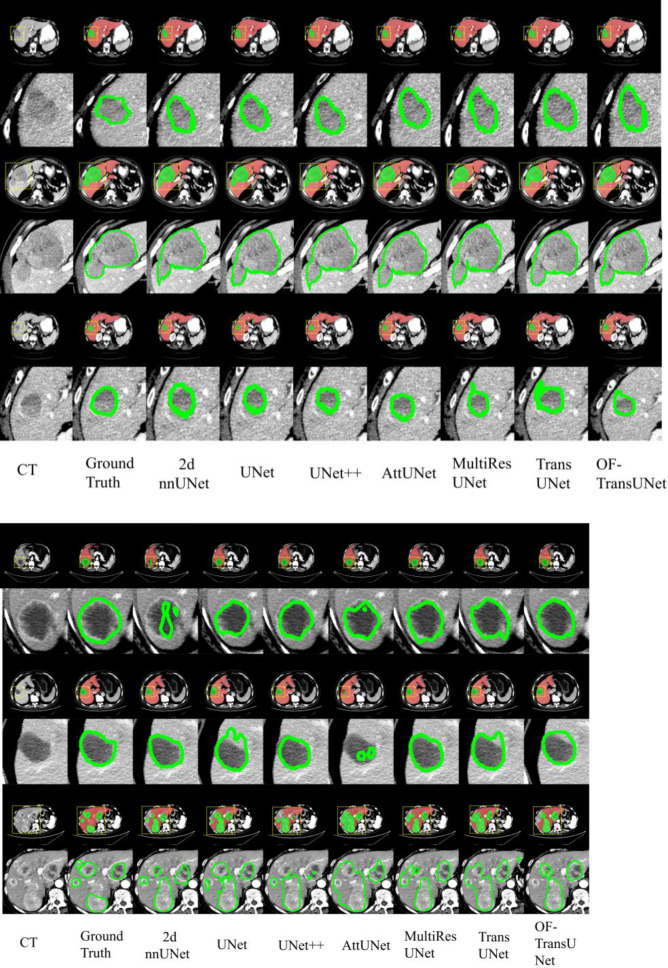
Qualitative comparison of segmentation results across different models on representative internal and external cases, including challenging small-lesion and multifocal-tumor scenarios. (**a**) Multimodality annotated hepatocellular carcinoma dataset, (**b**) representative cases (*n* = 42) from the external validation dataset

Figure 5 provides a visual comparison of the segmentation results from different models on representative cases from both the internal and external datasets. The images highlight instances where OF-TransUNet correctly identifies and delineates tumor boundaries, particularly for challenging, low-contrast lesions, while other models may produce fragmented or incomplete segmentations.

Whole-slice CT views are shown together with corresponding region-of-interest enlargements to facilitate visual comparison of tumor-boundary delineation across models. Yellow dashed boxes indicate the zoomed regions. Liver masks are shown in red, and tumor masks are shown as green fill with green boundary. The figure includes representative cases with small lesions and multiple tumors to illustrate model behavior under clinically challenging conditions. These examples are intended as qualitative illustrations only and should not be interpreted as substitutes for the cohort-level quantitative results reported in Tables 2–3.

## Discussion

### Principal findings

Our re-verified primary endpoint analysis showed a numerically higher per-patient tumor Dice for OF-TransUNet than for the nnU-Net baseline (mean paired difference +0.0388; 95% CI 0.0029 to 0.0748). However, because the paired differences deviated from normality, the pre-specified Wilcoxon signed-rank test was retained and yielded a borderline result (*p* = 0.0553), whereas a supplementary paired t-test reached nominal significance (*p* = 0.0347). The patient-level Dice result should therefore be interpreted as directionally favorable but not conclusively positive under the pre-specified primary analysis.

By contrast, the pre-defined medium-sized (10–50 mm) lesion analysis showed a clearer and statistically supported signal, with detection rate increasing from 0.190 to 0.286 at the pre-specified 10% threshold (McNemar exact *p* = 0.021) and an adjusted GEE odds ratio of 1.97 (95% CI 1.15–3.35; *p* = 0.013). This lesion-level advantage remained directionally consistent in post hoc sensitivity analyses using 5 and 15% overlap thresholds. Taken together, these findings suggest that, in this cohort, the strongest supported signal of benefit lies at the lesion-detection level rather than as definitive confirmation of superiority on the pre-specified patient-level primary endpoint.

### Benefit-to-complexity ratio and practical rationale

The observed gain relative to the added architectural and training complexity warrants explicit consideration. We interpret the practical value of OF-TransUNet not as a large-scale redesign, but as evidence that a highly localized Transformer augmentation can yield directionally favorable tumor-segmentation gains while largely preserving the deployment profile of the baseline 2D nnU-Net. The computational overhead remained modest (+8.4% parameters and +18.3% FLOPs), with no measurable latency penalty and only minimal peak-memory increase. Under this interpretation, the proposed design occupies a distinct point on the accuracy–efficiency spectrum: it does not aim to compete with substantially heavier 3D or multi-block hybrid systems on maximal benchmark performance, but rather to test whether limited contextual augmentation can produce practical gains without abandoning the simplicity and deployability of the original pipeline. We acknowledge that the mean paired Dice improvement in the external cohort (+0.0388) is modest in absolute terms; the primary justification for the proposed modification therefore rests on the controlled architectural attribution it enables and the statistically supported lesion-level detection gain rather than on a large absolute Dice increment.

### Relationship to prior work and architectural innovation

Recent Transformer–CNN hybrids often introduce substantial parameter growth and optimization complexity. Standard TransUNet variants typically embed deep Transformer cascades at the lowest resolution [2, 23]. In our re-verified external benchmark (Table 2), vanilla TransUNet did not outperform the nnU-Net baseline on per-patient tumor Dice (0.256 vs 0.240), and its paired comparison versus nnU-Net was not significant under the corresponding exploratory Wilcoxon analysis. This pattern is consistent with the possibility that, in our setting, heavier Transformer integration may increase optimization difficulty in limited medical datasets, particularly without extensive pre-training. On the other hand, lightweight hybrid or MLP-based architectures achieve efficiency by entirely redesigning the network [8, 15]. While parameter-efficient, such sweeping redesigns depart from the robust feature extraction pipeline of nnU-Net [3], making it more difficult to attribute observed performance differences specifically to the attention component rather than to broader architectural redesign.

The primary architectural contribution of OF-TransUNet is its highly constrained and causally isolatable integration strategy. By inserting only a single convolutional Transformer block at the mid-level encoder stage (Stage 3) of an otherwise unchanged nnU-Net, we preserve the baseline pipeline while introducing additional contextual modeling with limited architectural disruption. Furthermore, whereas standard TransUNet variants often rely on heavier token- or linear-projection-based Transformer modules, our inserted block remains convolutional throughout, retaining inductive bias while still modeling longer-range contextual interactions. This restrained architectural design is compatible with the favorable efficiency profile reported in Table 4 (+8.4% parameters) and with the directionally improved external tumor segmentation results observed for OF-TransUNet. Furthermore, our internal ablation (Table 1) showed that the evaluated immediate full fine-tuning configuration in the pooled benchmark led to complete failure (tumor Dice: 0.0000). However, additional repeated runs, including seed-controlled reruns, indicated that the broader issue was not deterministic collapse under every initialization, but unstable and poorly reproducible optimization behavior under immediate full fine-tuning. Across runs, outcomes ranged from near-zero collapse or severe degradation to only modest convergence, whereas both Simple Progressive and Output-Focused staged adaptation yielded stable performance. Together, these observations support staged unfreezing as a more reliable adaptation strategy for the inserted Conv-Transformer. In the re-verified external benchmark, this minimalist architecture achieved the highest mean per-patient tumor Dice among the tested models, although the pre-specified Wilcoxon comparison versus nnU-Net remained borderline rather than definitively positive. Taken together with the internal ablation results, these findings support cautious interpretation of a strategically placed, minimally invasive single-block design as a controlled way to test whether limited Transformer augmentation can provide practical benefit without large-scale architectural disruption.

Recent hybrid CNN–Transformer approaches on LiTS have reported tumor Dice values ranging from approximately 0.71 to 0.84 under varied experimental protocols [36, 38], and multi-encoder benchmarking studies have highlighted that reported gains may reflect differences in training schedules and split strategies rather than architecture alone [37]. These observations reinforce the rationale for our controlled within-pipeline comparison design.

## Design rationale and methodological considerations

Two design choices were central: (1) mid-level (stage 3) insertion to balance spatial resolution and semantic abstraction, and (2) staged Transformer adaptation to mitigate optimization instability. Internal ablation results supported this rationale. In particular, the immediate full fine-tuning configuration summarized in the pooled internal benchmark resulted in complete failure, whereas both Simple Progressive and Output-Focused schedules recovered stable performance. Additional repeated runs, including seed-controlled reruns, further showed that immediate full fine-tuning was unstable and poorly reproducible rather than uniformly successful, reinforcing the rationale for staged adaptation. The Simple Progressive strategy used a 70-epoch frozen stabilization phase followed by sequential release of one predefined Transformer parameter group every 20 epochs, with release onsets at epochs 70, 90, 110, and 130. In the final OF-TransUNet schedule, the inserted Transformer was frozen during the early stabilization phase and output-side components began to be released at epoch 90. The first released parameter group corresponded to [“final”], i.e., the output-side mapping component of the inserted Conv-Transformer block, followed by [“final”, “conv2”, “conv3”] at epoch 110, [“qkv”, “proj”] at epoch 130, and full unfreezing at epoch 150 and thereafter. A short Transformer-specific learning-rate warmup was applied during epochs 90–94. Repeated NaN/Inf events were still observed during later adaptation, indicating that output-focused unfreezing should be viewed as a practical stabilization strategy rather than a complete solution to optimization instability.

## Clinical significance and potential impact

Although the absolute mean paired Dice difference was modest (+0.0388), small overlap gains may still be relevant in low-contrast lesion settings if they reflect recovery of previously missed or fragmented tumor regions. In the present study, however, the clinically more interpretable signal came from the pre-defined medium-lesion detection analysis, which showed a 50.0% relative increase in detection rate (0.190 to 0.286). The persistence of this directional advantage under a stricter 15% overlap threshold argues against the result being driven solely by minimal near-threshold overlap events. The proposed modification also preserved a lightweight deployment profile, with only modest computational overhead and no measurable inference latency penalty. However, we did not directly measure downstream clinical outcomes such as radiologist correction time, treatment eligibility, staging impact, or longitudinal volumetric consistency; these translational implications should therefore be considered plausible but not demonstrated in the present study.

## Statistical interpretation

The statistical picture was mixed across endpoint levels. At the lesion level, the pre-specified 10% threshold analysis supported improved detection of medium-sized lesions by both McNemar and GEE methods, and post hoc threshold sensitivity analyses preserved the direction of effect across 5, 10, and 15% definitions. At the patient level, OF-TransUNet showed a positive mean paired tumor Dice difference, but the pre-specified Wilcoxon analysis was borderline (*p* = 0.0553) after non-normal paired differences were confirmed by the Shapiro-Wilk test. The supplementary paired t-test provides supportive but not primary evidence. Overall, this pattern is more consistent with a heterogeneous effect distribution across patients than with a uniform improvement across the entire cohort.

## Relationship between qualitative visualization and cohort-level quantitative metrics

In liver tumor segmentation, qualitative visualizations and cohort-level quantitative averages may convey different aspects of model performance and should be interpreted jointly rather than in isolation. Overlap-based measures such as Dice are highly sensitive to lesion size: a visually plausible contour on a very small lesion may still yield a low score if only a limited number of voxels are mismatched. This effect is amplified in heterogeneous cohorts containing low-contrast tumors, multifocal disease, and small-volume lesions. Consistent with the LiTS benchmark analysis [35], tumor segmentation performance is known to deteriorate substantially in small lesions and in cases with low tumor-to-liver contrast. Therefore, representative qualitative examples may visually demonstrate boundary recovery or lesion localization that is clinically meaningful, while the corresponding cohort-averaged overlap gain remains modest. Both forms of evidence are provided in the present study and should be considered complementary rather than contradictory.

## Limitations

Our study has several limitations that warrant careful consideration.

First, the external validation cohort was relatively small (*n* = 42), single-center, retrospectively collected, and acquired on a single scanner platform, limiting generalizability across acquisition protocols and institutions. In the re-verified patient-level analysis, the mean paired Dice difference was +0.0388 (95% CI 0.0029 to 0.0748), but the pre-specified Wilcoxon test remained borderline (*p* = 0.0553), underscoring the fragility of inference in a small and heterogeneous cohort.

Second, we did not perform a formal quantitative domain-shift analysis between the public development dataset and the external cohort (e.g., intensity-distribution comparison or scanner-stratified feature analysis). Because the public dataset included multi-vendor acquisitions whereas the external cohort was collected on a single GE platform, scanner- and protocol-related shifts may have influenced both baseline and Transformer-augmented contextual modeling. We also did not conduct a leave-one-center-out experiment within the public dataset, which is acknowledged as a limitation for assessing domain generalization.

Third, while OF-TransUNet showed a numerically higher tumor HD95 than nnU-Net (104.353 vs 96.776 mm; approximately +7.6 mm), the interpretation of this increase remains limited. The aggregate pattern suggests a possible recall–boundary trade-off, but the present study does not provide sufficient evidence to attribute the HD95 shift primarily to true-positive satellite nodule recovery. As a descriptive post hoc paired case-level summary among cases with valid tumor HD95 in both models (*n* = 39), HD95 worsened in 25 cases (64.1%), including increases greater than 5 mm in 18 cases (46.2%) and greater than 10 mm in 11 cases (28.2%), while improvement was observed in 14 cases (35.9%). This pattern indicates heterogeneity of boundary effects across cases rather than uniform deterioration, but it remains descriptive and non-confirmatory. The available qualitative review was small in scale and post hoc, and should therefore be interpreted as hypothesis-generating rather than confirmatory. We also did not perform a paired inferential analysis specifically for tumor HD95, because invalid or undefined values from empty predictions led to model-dependent effective sample sizes.

Fourth, although the output-focused progressive unfreezing schedule improved stability relative to immediate full fine-tuning, repeated NaN/Inf events were still observed during Transformer adaptation in the final training logs. Thus, the proposed schedule should be understood as a practical mitigation strategy rather than a complete solution to optimization instability. While we added seed-controlled reruns to examine the behavior of immediate full fine-tuning, we did not perform a formal large-scale multi-seed robustness study; therefore, our conclusion should be interpreted as evidence of unstable and poorly reproducible optimization rather than proof of deterministic collapse under all initializations.

Fifth, although overlap-threshold sensitivity analyses (5 and 15%) were added post hoc and supported directional robustness of the medium-lesion finding, they were not pre-specified and therefore do not alter the original inferential hierarchy of the study.

Finally, the study focused on technical segmentation performance rather than direct clinical workflow outcomes. We did not measure radiologist correction time, staging impact, treatment eligibility changes, or longitudinal volume consistency. These clinically meaningful endpoints require dedicated prospective evaluation.

## Future work

Future work should more directly target the observed tumor HD95 trade-off through boundary-aware optimization, for example by incorporating contour-sensitive or surface-distance-aware loss functions, lesion-wise false-positive suppression, and boundary-focused post-processing designed to preserve small-lesion recall while reducing distant spurious components.

Future work should prioritize multi-center and adequately powered external validation, with pre-specified size-stratified lesion analyses, boundary-focused evaluation, and more formal assessment of domain generalization. Additional directions include systematic exploration of alternative insertion locations, shallow multi-block variants, lightweight 2.5D/3D extensions, and prospective reader-in-the-loop studies to assess clinical workflow impact, interpretability, and correction burden.

## Conclusion

We present a restrained modification of nnU-Net that integrates a single Conv-Transformer block with an output-focused progressive unfreezing design. In this single-center external validation cohort, the approach showed a numerically higher per-patient tumor Dice and a clearer signal in the pre-defined medium-sized lesion detection analysis, while adding only modest computational overhead (+8.4% parameters, +18.3% FLOPs, and approximately 1 MB peak memory) without measurable inference latency penalty. Because the pre-specified non-parametric patient-level primary analysis was borderline and did not reach conventional significance, the tumor Dice finding should be interpreted cautiously and not as definitive evidence of superiority. Taken together, the lesion-level findings, controlled architectural design, and lightweight deployment profile support further multi-center evaluation and boundary-focused refinement of this minimally invasive augmentation strategy.

## Electronic supplementary material

Below is the link to the electronic supplementary material.

Supplementary material 1

## Data Availability

The public HCC-TACE dataset used for internal development is openly accessible at 10.1038/s41597-023-01928-3 [4]. The external validation cohort is not publicly released due to institutional data-sharing policies. De-identified derived results may be shared upon reasonable request, subject to institutional approval and applicable data-sharing restrictions. Requests should be directed to the corresponding author, Xiaoping Pan (pxp74@sina.com).

## Abbreviation

AMP: Automatic Mixed Precision
CI: Confidence Interval
CNN: Convolutional Neural Network
CT: Computed Tomography
DSC: Dice Similarity Coefficient
GEE: Generalized Estimating Equations
HCC: Hepatocellular Carcinoma
HCC-TACE: Hepatocellular Carcinoma – Transarterial Chemoembolization (public dataset)
HD95: 95th Percentile Hausdorff Distance
HU: Hounsfield Unit
IoU: Intersection over Union
nnU-Net: no-new-U-Net
OF-TransUNet: Output-Focused TransUNet
OR: Odds Ratio
SD: Standard Deviation
ViT: Vision Transformer
VS: Volume Similarity
FLOPs: Floating-Point Operations

## Code availability

A public repository for OF-TransUNet has been created at https://github.com/callhe-ai/OF-TransUNet. During peer review, the repository includes the core trainer implementation (`nnUNetTrainerTransUNet.py`), repository-level documentation, dependency information, and supplementary implementation notes describing the staged Transformer unfreezing schedules. The core implementation is therefore already publicly available for transparency during peer review, while complete training and inference scripts, evaluation scripts, pretrained weights, and full reproducibility documentation will be finalized and expanded upon manuscript acceptance. The repository will also include step-by-step instructions for reproducing the principal quantitative results reported in Tables 2–3.
