# Supplementary material for: Efficient transformer integration in nnU-Net for liver tumor segmentation: an external validation study
Source: BMC Med Imaging. 2026 May 22;26:356. doi: 10.1186/s12880-026-02406-z (PMC13374201; doi:10.1186/s12880-026-02406-z)
Supplement: Supplementary file 1 — Supplementary material 1 [file 12880_2026_2406_MOESM1_ESM.docx]

**SUPPLEMENTARY MATERIAL**

**Supplementary Table S1. Sensitivity analysis of medium-sized lesion (10–50 mm) detection under alternative overlap thresholds**

| **Overlap threshold** | **nnU-Net rate (95% CI)** | **OF-TransUNet rate (95% CI)** | **Absolute diff** | **McNemar exact p** | **GEE OR (95% CI)** | **GEE p** |
| --- | --- | --- | --- | --- | --- | --- |
| 5% | 0.226 (0.150–0.326) | 0.286 (0.200–0.390) | +0.060 | 0.227 | 1.51 (0.92–2.46) | 0.101 |
| 10% | 0.190 (0.121–0.287) | 0.286 (0.200–0.390) | +0.095 | 0.021 | 1.97 (1.15–3.35) | 0.013 |
| 15% | 0.155 (0.093–0.247) | 0.262 (0.180–0.365) | +0.107 | 0.035 | 2.20 (1.16–4.16) | 0.016 |

**Notes:** Detection was redefined post hoc using overlap thresholds of 5%, 10%, and 15% within the same 10–50 mm lesion subset (n = 84). Detection rates are reported with Wilson 95% confidence intervals. McNemar p-values are exact paired p-values. GEE odds ratios were adjusted for lesion diameter and patient-level clustering. These analyses were supportive robustness checks and were not pre-specified.

**──────────────────────────────────────────────────**

**Supplementary Note S1. Definition of staged Transformer unfreezing schedules**

To improve implementation transparency, we summarize here how the staged unfreezing schedules were defined in the internal development experiments.

**No Fine-tuning**

The inserted Conv-Transformer remained frozen throughout training, and only the nnU-Net backbone and decoder were updated.

**Full Fine-tuning**

All Transformer parameter groups were trainable from the beginning of training, without any frozen stabilization phase.

**Simple Progressive**

In the Simple Progressive schedule, the inserted Transformer was partitioned into four predefined parameter groups in the implementation and released sequentially in the default order [1, 2, 3, 4]. The Transformer remained frozen for epochs 0–69, after which one parameter group was released every 20 epochs, corresponding to release onsets at epochs 70, 90, 110, and 130.

**Output-Focused (final)**

In the final implemented OF-TransUNet schedule, the Transformer remained frozen through epochs 0–89. At epoch 90, the output-side mapping component (["final"]) was released first, and the optimizer was reconfigured to include the newly trainable parameters. A short Transformer-specific warmup was then applied during epochs 90–94.

Additional parameter groups were released at epoch 110 (["final", "conv2", "conv3"]) and epoch 130 (["qkv", "proj"]), followed by full unfreezing at epoch 150 and thereafter.

**Interpretive note**

The output-focused schedule should therefore be understood as an explicitly implemented output-prioritized staged adaptation strategy, in which output-proximal Transformer components were released before deeper internal components. The epoch boundaries and release groups reported here are consistent with the revised main manuscript and the publicly available core trainer implementation provided during peer review.

**──────────────────────────────────────────────────**

**Supplementary Table S2. Descriptive paired case-level summary of tumor HD95 changes between OF-TransUNet and nnU-Net**

| **Measure** | **Value** |
| --- | --- |
| Paired valid tumor HD95 cases | 39 |
| Worsened HD95 (>0 mm) | 25/39 (64.1%) |
| Worsened HD95 >5 mm | 18/39 (46.2%) |
| Worsened HD95 >10 mm | 11/39 (28.2%) |
| Improved HD95 (<0 mm) | 14/39 (35.9%) |
| Improved HD95 <-5 mm | 10/39 (25.6%) |
| Improved HD95 <-10 mm | 8/39 (20.5%) |
| Absolute change ≤10 mm | 20/39 (51.3%) |

**Notes**

Only cases with valid tumor HD95 in both models were included. This table is descriptive and post hoc, and is intended to contextualize the aggregate HD95 shift rather than provide a new confirmatory endpoint.

**Supplementary Table S3. Representative published LiTS liver tumor segmentation results for contextual reference only**

*Direct numerical comparison across studies is not appropriate due to substantial heterogeneity in experimental protocols (see table note). Values are provided for descriptive contextual background only.*

| **Study** | **Year / Journal** | **Method type** | **Reported tumor metric** | **Key protocol notes** |
| --- | --- | --- | --- | --- |
| Bilic et al. (LiTS Benchmark) [35] | 2023 / Medical Image Analysis | Official challenge benchmark (mixed submissions) | Best tumor Dice: 0.674 (ISBI 2017), 0.702 (MICCAI 2017), 0.739 (MICCAI 2018) | Official hidden test set (70 cases); challenge conditions; ensemble and post-processing permitted; not reproducible locally |
| Shin et al. (G-UNETR++) [36] | 2026 / Diagnostics | 3D hybrid CNN-Transformer (gradient-enhanced, two-stage pipeline) | Tumor DSC: 0.844 ± 0.078 | Custom split (97/20/20); 3D volumetric; two-stage liver-then-tumor pipeline; morphological dilation post-processing |
| Sushma & Mitra (LiT-HiSegFormer-Net) [37] | 2026 / Intelligence-Based Medicine | 2D CNN + Swin Transformer hybrid (custom split) | Tumor Dice per-case: 72.65%; Global Dice: 82.75% | Custom split (40 train / 5 val / 35 test); 2D slice-based; no ensemble; authors note results not directly comparable to official leaderboard |
| Ye et al. (Multi-encoder U-Net benchmarking) [38] | 2026 / Biomedical Engineering | Multi-backbone 2D U-Net family (VGG16/19, ResNet, MobileNet); 3-fold CV | Best tumor Dice: 92.09% (VGG16-UNet, 100 epochs) | 3-fold cross-validation on 131 labeled cases; 2D slice-based; authors note gains may reflect training schedule differences rather than architecture alone |
| Chen et al. (SBM–Attention U-Net) [39] | 2026 / Sensors | 2D hybrid attention network (BiFormer + SCDA + MSB) | Tumor Dice per-case: 82.30%^a^ | Custom 6:2:2 split; 2D slice-based; mean Dice reported in abstract (0.9257) includes both liver and tumor classes and is not tumor-only |
| **This study — supplementary LiTS fold_0** | **2026 / Present manuscript** | **2D nnU-Net v2 pipeline (controlled within-pipeline comparison)** | **OF-TransUNet: 0.5325 ± 0.3049 vs baseline: 0.5012 ± 0.3265 (mean diff +0.0313; Wilcoxon p = 0.0339)** | **Single pre-fixed fold (fold_0; n = 27 validation cases); 131 public annotated cases only; no hidden test access; supplementary contextual evidence only; not a definitive LiTS-wide estimate** |

**Table notes:**

^a^ SBM–Attention U-Net: the mean Dice of 0.9257 reported in the abstract of Chen et al. [39] refers to the mean across liver and tumor classes combined; the tumor-specific per-case Dice of 82.30% is taken from Table 2 of that paper under a 6:2:2 custom split.

**Direct numerical comparison across published LiTS studies is not appropriate** because of substantial heterogeneity in the following dimensions:

- (1) Train/validation/test split strategy (custom fixed splits vs. cross-validation vs. official hidden test evaluation);
- (2) Use of public annotated cases versus official hidden test set (70 cases with non-public labels);
- (3) Model dimensionality (2D slice-based vs. 3D volumetric);
- (4) Use of cascaded pipelines, ensembling, test-time augmentation, or post-processing;
- (5) Reported metric definitions (per-case Dice, global Dice, DSC, lesion-level detection metrics);
- (6) Training duration, optimizer settings, and preprocessing pipelines.

This table is therefore provided for **descriptive contextual reference only**. The primary architectural evidence in the present study remains the controlled within-pipeline comparison on the independent external validation cohort (Tables 1–3 in the main manuscript). The supplementary LiTS fold_0 result from the present study is interpreted as supportive contextual evidence rather than a definitive LiTS-wide performance estimate.

Abbreviations: DSC, Dice similarity coefficient; CV, cross-validation; 2D, two-dimensional; 3D, three-dimensional.
